# The timing and quality of antenatal care received by women attending a primary care centre in Iquitos, Peru: A facility exit survey

**DOI:** 10.1371/journal.pone.0229852

**Published:** 2020-03-05

**Authors:** Sara Jabeen Wynne, Rui Duarte, Gilles de Wildt, Graciela Meza, Abi Merriel

**Affiliations:** 1 Medical School, College of Medical and Dental Sciences, University of Birmingham, Birmingham, United Kingdom; 2 Liverpool Reviews and Implementation Group, University of Liverpool, Liverpool, United Kingdom; 3 Institute of Clinical Sciences, College of Medical and Dental Sciences, University of Birmingham, Birmingham, United Kingdom; 4 Faculty of Medicine, National University of the Peruvian Amazon, Punchana, Peru; 5 Bristol Medical School, Population Health Sciences, Southmead Hospital, University of Bristol, Bristol, United Kingdom; University of Oxford, UNITED KINGDOM

## Abstract

**Background:**

Maternal mortality is high in Loreto, Peru, but can be reduced by high quality antenatal care. Indicators for the quality of antenatal care received include the timing (with respect to gestational age) and number of antenatal appointments attended, the delivery of antenatal services and health information, and women’s perceptions about their care. This study investigated these indicators amongst women receiving antenatal care in predominantly the *San Juan Bautista* district of Iquitos, Loreto. This was to identify areas for improvement through comparison with antenatal guidelines published by the Ministry of Health, Peru, and the World Health Organization.

**Methods:**

A total of 134 women were recruited at the *Centro de Salud*, *San Juan*—a primary care centre in Iquitos. Information about the delivery of antenatal services and the number of and gestational ages at appointments attended was collected from 121/134 women’s hand-held antenatal cards. The delivery of health information and women’s perceptions about their antenatal care were investigated through questionnaires (133/134 completed). Descriptive statistics, such as frequencies and valid percentages, were determined.

**Results:**

Hand-held antenatal cards revealed that 52.9% of participants began their antenatal care in the first trimester. Compared to national guidelines, 42.1% attended appointments at recommended gestational ages and no women received all recommended antenatal services. Most women received information about identifying complications in pregnancy and health and lifestyle topics. Over 85% of women reported satisfaction with their antenatal care.

**Conclusions:**

Timely antenatal attendance and delivery of services should be encouraged to meet national and global standards. Although all services were not delivered in a combined manner according to national guidelines, individual services were mostly delivered to a high standard and therefore a high proportion of women were satisfied with their antenatal care.

## Background

Maternal mortality remains an important global health issue. Over 99% of the 303,000 maternal deaths which occurred due to complications of pregnancy and childbirth in 2015 were in developing countries [1]. One contributing factor to this is poor quality antenatal care (ANC) [2–4]. High quality universal ANC is essential to reduce maternal mortality [1]. ANC requires clinicians to monitor the health of pregnant women, identify and manage risks, and provide health education to prevent maternal and neonatal morbidity and mortality from treatable causes such hypertensive disorders of pregnancy and diabetes [1, 5–9]. The World Health Organization (WHO) updated its antenatal guidelines in 2016 to include changes such as increasing the recommended number of antenatal contacts from four to eight [1]. As these guidelines are disseminated, it is increasingly important to assess the delivery of ANC in areas with high maternal mortality ratios (MMR), particularly in developing regions of the world, to maximise the quality of ANC received [1]. One such region is Loreto within the Peruvian Amazon (MMR: 119.5 maternal deaths per 100,000 live births) [10, 11]. Previous literature has demonstrated that the remoteness and lack of accessibility of this region by road has resulted in less effective healthcare being received in this region compared to mainland Peru [12].

Indicators of ANC quality include attending the first appointment in the first trimester to allow for early risk management and attending an adequate number of appointments to ensure the delivery of appropriately timed antenatal services [1, 13–15]. Only two studies have investigated these indicators in Loreto. One study observed that in the regional capital of Iquitos, 32.5% of 2647 women initiated their ANC in their first trimester. Another study reported that an average of six antenatal appointments were attended during the pregnancies of 3191 postpartum women in Iquitos—the recommended minimum by the Ministry of Health, Peru (MoHP) [16–18]. Whether the appointments were attended within the MoHP recommended gestational ages was not clear.

Attending antenatal appointments alone does not guarantee good quality ANC. Previous literature from 91 low and middle-income countries has shown that almost a third of women who access ANC do not receive at least three antenatal services, such as blood pressure measurements and blood tests [2]. These services, as well as health education, must also be sufficiently received to maximise the quality of ANC [19]. The Peruvian Demographic and Family Health Survey (2015) reported that over 90% of 520 women from Loreto had their blood pressure measured during their ANC for their most recent pregnancies [6]. However, there were no reports for the delivery of health information or other MoHP recommended services, e.g. urinary protein measurements [18].

Additionally, as part of the quality assurance of ANC, the WHO recommends assessing antenatal services according to women’s attitudes and needs by investigating their perceptions about their ANC. This is useful for understanding the uptake of ANC [20–22]. The new WHO guidelines are also informed by research concerning women’s perceptions, in addition to clinical evidence, to promote a ‘positive pregnancy experience’ [1]. There is limited literature concerning women’s perceptions about their ANC in Loreto. One qualitative study amongst twenty post-partum women in Iquitos found that some women were content with their care, whilst others reported that they wanted more information and appointments during their ANC [23].

## Methods and materials

### Objectives

This study aimed to expand the limited research base concerning indicators of the quality of ANC received by women in Loreto. These indicators include women’s antenatal attendance, the delivery of antenatal services and health information, and women’s perceptions about their ANC. Quality of ANC was investigated amongst a population of women from predominantly the *San Juan Bautista* district of Iquitos, Loreto. This was to identify areas for improvement within the ANC received through comparison with the expected standards of the MoHP and new WHO antenatal guidelines.

### Study design

A cross-sectional exit survey.

### Setting

Maternal mortality continues to be a public health concern in the Peruvian region of Loreto, with the regional MMR being almost double that of the national MMR of Peru (68 per 100,000 live births) [10, 24]. For this reason, the regional government of Loreto declared maternal health research a priority for 2015–2021 [25]. Consequently, the city of Iquitos was chosen as the study area as it is the capital of Loreto. The main language spoken in Loreto is Spanish (92.6%), with other languages including Quechua (0.7%), Sordomudo (0.1%) and native Amazonian languages (6.4%). Just under a third (63.9%) of the population is employed in Loreto [26].

Iquitos is located in north-eastern Peru, within the Amazon basin, and is surrounded by the Amazon, Nanay and Itaya rivers. It is the ninth most populated city of Peru (471,993) and is split into four districts: *Iquitos*, *San Juan Bautista*, *Punchana* and *Belen* [27]. The predominant ethnic group in Iquitos is *Mestizo* (89.8%), with others including White (3.8%), Afro-Peruvian (3.1%) and Quechua (1.8%) [27]. The literacy rate is 92.6% [27].

Data for this study were collected during February 2017 at the Centro de Salud, San Juan (CSSJ)–a primary care centre in Iquitos. This healthcare centre was selected as it is one the largest and busiest healthcare centres in Iquitos, and the leading provider of primary healthcare within the *San Juan Bautista* district of the city. Other healthcare centres include the *Moronacocha*, *6 de Octubre and Bellavista Nanay* centres, which are the lead providers in the *Iquitos*, *Belen and Punchana* districts, respectively. Other centres could not be visited due to the time and financial constraints of this study. The CSSJ provides healthcare within the fields of general medicine, paediatrics, care of the elderly, psychiatry, infectious diseases, sexual health, maternal health and dentistry. All appointments and services are free of charge under the MoHP funded *Seguro Integral de Salud* government health programme or the *EsSalud* programme, which is organised by various employers [28].

Approximately one-thousand women attend the CSSJ every year for routine antenatal appointments, where antenatal care is provided by obstetricians, nurses and midwives. Following routine appointments, women are responsible for seeking antenatal services which are not offered within these appointments, such as immunisations, laboratory and ultrasound investigations. Women attending routine appointments at other clinics may access these services at the CSSJ, and vice versa. A history of each woman’s ANC is recorded in hand-held antenatal cards designed by the MoHP, which allows women access to institutional deliveries. Vaginal delivery services are also offered at the CSSJ, with approximately 650 women giving birth at this centre every year.

### Participants

Women attending the CSSJ for either a routine or ultrasound appointment as part of their ANC were considered for participation if they met the following selection criteria:

### Inclusion criteria:

- ≥ 19 years. The local research ethics committee did not permit the inclusion of women below age of 19, as this is considered the adult age with respect to ANC in Peru [18]. An upper age limit was not set in order to include all adult women.
- Spanish speaking.
- Capable of giving informed consent.

### Exclusion criteria:

- Women attending for emergency ANC on the day of potential recruitment.
- Women acquainted with the study interpreters (to respect confidentiality).

Women were provided with information about the study, with the assistance of an interpreter, and provided their written, informed consent take part.

### Study size

Due to the time constraints of this study, the maximum number of eligible and consenting women were recruited in the time available.

### Data measurement

#### Case notes extraction

After appointments, data were collected from women’s hand-held antenatal cards for their current pregnancies and noted in a checklist developed for this study based on MoHP guidelines. The MoHP recommends services to be provided within six gestational windows of pregnancy, which coincide with the recommended number of six appointments: <14 weeks, 14–21 weeks, 22–24 weeks, 25–32 weeks, 33–36 weeks and 37–40 weeks [18]. The checklist comprised sections for noting the number of routine appointments attended, the gestational ages at routine appointments attended and the antenatal services received. Participants’ characteristics (current gestational age and obstetric medical histories) were also noted in the checklist (gestational ages were calculated from the last menstrual period in women’s hand-held antenatal cards and this method was adopted for this study). These data were collected by the first author.

#### Client interview

A structured, non-validated questionnaire was developed which consisted of three sections: delivery of information, perceptions about ANC and participant characteristics [S1 Appendix].

1. *Delivery of information*. Structured questions were used to investigate the delivery of MoHP recommended information topics. This included six signs of complications of pregnancy (intense and persistent headache, fever, vaginal bleeding, fluid leaking from the vagina, reduction or absence in fetal movements and premature contractions before 37 weeks of pregnancy) and six health and lifestyle topics (diet and nutrition, sexual health, family planning, breastfeeding, alcohol intake and smoking during pregnancy) [18].
2. *Perceptions about ANC*. Women’s perceptions were investigated using structured questions adapted from a WHO questionnaire and other literature [29–31]. Women who reported to have received information about the six health and lifestyle topics were asked whether they were content with the information received, or would prefer more or less information. Women’s perceptions about their routine appointments (frequency, duration and privacy experienced), the usefulness of their hand-held antenatal cards and any written information received were also investigated. One question assessed overall satisfaction with ANC using a five-point Likert scale, emulating a question in a previously validated WHO questionnaire which asked women to rate their overall satisfaction with their ANC using a three-point Likert scale [29].
3. *Participant characteristics*. A combination of structured and semi-structured questions were used to investigate participants’ age, marital status, employment status, educational level, gravidity, parity and history of miscarriage.

The questionnaires were translated into Peruvian Spanish [S2 Appendix] and administered after ANC appointments as interviews with an interpreter. The interpreters had received training about the questionnaire and study more generally. Ten interviews were conducted as part of the external pilot, where it was determined that delivering questionnaires as interviews reduced the likelihood of women missing questions.

### Bias

Interviews were conducted with auditory privacy from the healthcare providers to minimise the risk of their presence affecting responses [19]. Women were also assured that their responses would remain anonymous to minimise the effects of social desirability bias on this study.

### Analysis

#### Statistical methods

The data were analysed using IBM SPSS Statistics (version 22.0). Frequencies were determined to calculate proportions with 95% confidence intervals. Medians and inter-quartile ranges (IQR) for non-normally distributed variables were also calculated. Missed responses were excluded from any analyses and participant totals were adjusted per variable to reflect this.

#### Quantitative variables

Data collected from hand-held antenatal cards were analyzed such that women who had attended the same number of appointments as gestational windows reached in their pregnancy were considered to have attended the number of appointments recommended by the MoHP. Those who had attended at least one appointment during each gestational window reached in their pregnancy were considered to have attended according to the MoHP schedule.

The delivery of antenatal services within routine appointments were analysed through direct comparison with the MoHP antenatal schedule. For example, if a woman attended an appointment between 22–24 weeks gestation, her hand-held antenatal card was examined for evidence of all services which should have been received within that appointment. Services received during routine appointments according to the MoHP antenatal protocol are outlined in Table 1 [18].

**Table 1:** Services received within routine appointments according to the MoHP antenatal schedule.

Service received	Gestational windows in which routine appointments are attended					
	<14 weeks	14–21 weeks	22–24 weeks	25–32 weeks	33–36 weeks	37–40 weeks
**Maternal weight measurement**	X	X	X	X	X	X
**Blood pressure measurement**	X	X	X	X	X	X
**Pulse measurement**	X	X	X	X	X	X
**Temperature measurement**	X	X	X	X	X	X
**Urinary protein investigation (dipstick)**	X	X	X	X	X	X
**Uterine fundal height measurement (manual)**		X	X	X	X	X
**Fetal heartbeat assessment**		X	X	X	X	X
**Fetal presentation assessment**				X	X	X
**Prescription for folic acid supplements**	X					
**Prescription for folic acid and iron supplements**		X	X	X	X	X
**Prescription for calcium supplements**			X	X	X	X

***X*** *= a service recommended by the MoHP within a gestational window*

To account for the fact that some antenatal services were not received on the same day as routine appointments, these services were analysed according to completed gestational windows. For example, if the MoHP recommended a service between 22–24 weeks, data collected from women with gestational ages ≥25 weeks were analysed for evidence of delivery of this service. This allowed for a fair assessment of the delivery of ANC by ensuring the entire window had passed before determining whether a service was received or not. Services to be received out of routine appointments according to the MoHP antenatal protocol are outlined in Table 2 [18].

**Table 2:** Services expected to be received out of routine appointments according to the MoHP antenatal schedule.

Service
**Women with gestational ages ≥14 weeks (completed window <14 weeks)**
Maternal height measured
Blood group determined
Rhesus group determined
Pelvic exam
Cervical smear
First haemoglobin measurement
First microscopic urine examination
First serum glucose measurement
First HIV status test
First syphilis status test
First ultrasound examination
**Women with gestational ages ≥22 weeks (completed window 14–21 weeks)**
Dental examination
First anti-tetanus immunisation
**Women with gestational ages ≥25 weeks (completed window 22–24 weeks)**
Second ultrasound examination
Second anti-tetanus immunisation
**Women with gestational ages** ≥**33 weeks (completed window 25–32 weeks)**
Second serum glucose measurement
**Women with gestational ages** ≥**37 weeks (completed window 33–36 weeks)**
Second haemoglobin measurement
Second microscopic urine examination
Second HIV status test
Second syphilis status test

Data were also analysed to evaluate how many women received all services exactly as per the MoHP protocol (i.e. every recommended service within the correct gestational window, out of gestational windows reached in their pregnancy).

### Ethics approval and consent to participate

Ethical approval was granted by the Internal Research Ethics Committee at the University of Birmingham, United Kingdom, on December 16th 2016 (reference number: Y16_C1_07) and from the Institutional Committee for Research Ethics of the Regional Directorate of Health, Loreto, on January 24th 2017 (reference number: 005-CIEI-DRSL-2017). Written, informed consent was gained from all study participants with the assistance of a study interpreter.

## Results

### Participants

**Fig 1:**
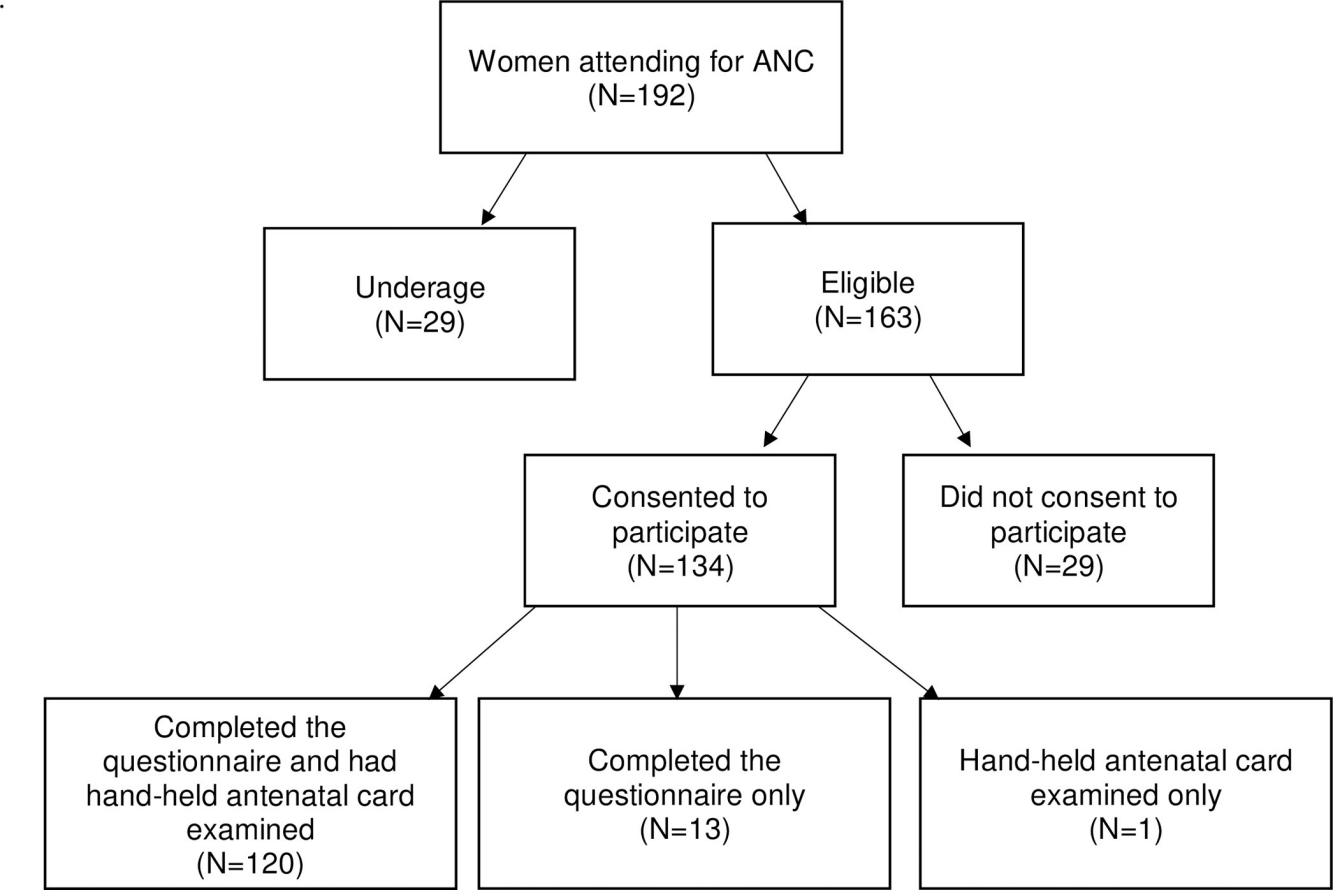
Recruitment of participants. **N** = number of women per category.

Fig 1 demonstrates the recruitment of participants in this study. One-hundred and ninety-two women attended the CSSJ for ANC during the study period. Twenty-nine of these women (15.1%) could not be recruited due to being underage. A total 134/163 eligible women consented to participate. Reasons for not participating included lack of time after appointments and not wishing to share experiences. Thirteen women out of the 134 participants (9.7%) only consented to complete the questionnaire. Reasons for not consenting to have their hand-held antenatal cards examined included the wish for privacy and forgetting their cards. One consenting woman left the clinic after her hand-held antenatal card was examined and did not complete the questionnaire. Overall, 133 questionnaires were completed and 121 hand-held antenatal cards were examined, out of which 120/134 (89.6%) women completed both the questionnaire and had their hand-held antenatal card examined.

The participants’ demographic and obstetric characteristics are presented in Table 3. Over one-quarter of participants received ANC at other healthcare centres in Iquitos. These included the *Progresso*, *America*, *Rumococha*, *Peña Negra* and *Villa Buen Pastor* centres in *San Juan Bautista*, the *6 de Octubre*, *Modelo* and *Cardozo* centres in *Belen*, the *Moronacocha* centre in *Iquitos*, the *Bellavista Nanay* and *1 de Enero* centres in *Punchana* and the Regional Hospital of Iquitos (the largest hospital in the city).

**Table 3:** Participants’ demographic and obstetric characteristics.

Characteristic	n %
**Age (years) (N = 133)**	
**Median (Q1-Q3):** 26 (22–30)	
19–24	60 45.1
25–34	58 43.6
35–42	15 11.3
**Marital status (N = 133)**	
Single, never married	7 5.3
Co-habiting with partner	95 71.4
Married	26 19.5
Separated	1 0.8
Widowed	2 1.5
Prefer not to say	2 1.5
**Educational level (completed) (N = 133)**	
Primary	55 41.4
Secondary	53 39.8
University or technical college	25 18.8
**Employment status (N = 131)**	
Employed	25 19.1
Unemployed	91 69.5
Student	15 11.5
**Trimester of pregnancy at time of recruitment (N = 121)**	
First *(<13 weeks of gestation)*	9 7.4
Second *(13–27 weeks of gestation)*	49 40.5
Third *(>27 weeks of gestation)*	63 52.1
**Gravidity (N = 132)**	
Primigravida	26 19.7
Multigravida	106 80.3
**Parity (N = 131)**	
**Median (Q1-Q3):** 1 (0–2)	
0	37 28.2
1–3	81 61.8
>3	13 9.9
**History of miscarriage (N = 131)**	
Yes	45 34.4
No	86 65.6
**Personal obstetric medical history (N = 121)**	
Pelvic/uterine surgery	9 7.4
Habitual/recurrent abortions	1 0.8
Pre-eclampsia	1 0.8
None	110 90.9
**Only attended routine antenatal appointments at CSSJ (N = 121)**	
Yes	85 70.2
No	36 29.8

**N** = number of women for which data were collected for each variable, **Q1-Q3** = quartile 1 –quartile 3

### Routine antenatal attendance

Table 4 presents women’s antenatal attendance, with respect to the number and gestational ages at routine appointments attended. The median gestational age at women’s first appointments was 11.5 weeks (Q1-Q3: 8–17) and just over half (52.9%) of the participants attended their initial appointment during their first trimester. The number of appointments attended ranged from a single appointment to 10, with a median of 4 (Q1-Q3: 2–6). Just under three-quarters (72.7%) of women attended the MoHP recommended number of appointments for their gestational age. Over half (57.9%) did not attend appointments according to the MoHP schedule.

**Table 4:** Participants’ routine antenatal attendance (N = 121).

Attendance feature	n % (95% CI)
**Timing of the first appointment (with respect to gestational age)**	
**Median gestational age in weeks (Q1-Q3):** 11.5 (8–17)	
First trimester	64 52.9 (44.0–61.6)
Second trimester	53 43.8 (35.3–52.7)
Third trimester	4 3.3 (1.3–8.2)
**Number of appointments attended**	
**Median (Q1-Q3):** 4 (2–6)	
1–3	56 46.3 (37.6–55.1)
4–6	49 40.5 (32.2–49.4)
7–10	16 13.2 (8.3–20.4)
**Attended MoHP recommended number of appointments for gestational age**	
Yes	88 72.7 (64.2–79.9)
No	33 27.3 (20.1–35.8)
**Attended appointments according to the MoHP schedule**	
Yes	51 42.1 (33.7–51.1)
No	70 57.9 (48.9–66.3)

**CI** = confidence interval, **Q1-Q3** = Quartile 1 –Quartile 3

### Delivery of antenatal services

The antenatal services received by women during routine appointments in each gestational window are presented in Table 5. All women had their weight, blood pressure, pulse and temperature measured in every gestational window in which they attended an appointment. Prescriptions for supplements (folic acid, iron and calcium) and uterine fundal height measurements were received by at least 90% or more of women attending during MoHP recommended windows for these services. The least delivered service was the investigation of urinary protein in women attending before 14 weeks (14.9%) and between 14–21 weeks (28.9%). Confidence intervals for these values, as well as the frequencies and proportions of women who did not receive antenatal services within routine appointments, are presented as additional information [S1 Table]. Two women out of 121 (1.7%, 95% CI: 0.5%– 5.8%) received all services delivered within routine appointments (as outlined in Table 5) exactly as per the MoHP protocol.

**Table 5:** Delivery of antenatal services during routine appointments.

Service received	Gestational windows in which routine appointments were attended					
	<14 weeks (N = 74)	14–21 weeks (N = 97)	22–24 weeks (N = 51)	25–32 weeks (N = 76)	33–36 weeks (N = 33)	37–40 weeks (N = 16)
	n %	n %	n %	n %	n %	n %
**Maternal weight measurement**	74 100.0	97 100.0	51 100.0	76 100.0	33 100.0	16 100.0
**Blood pressure measurement**	74 100.0	97 100.0	51 100.0	76 100.0	33 100.0	16 100.0
**Pulse measurement**	74 100.0	97 100.0	51 100.0	76 100.0	33 100.0	16 100.0
**Temperature measurement**	74 100.0	97 100.0	51 100.0	76 100.0	33 100.0	16 100.0
**Urinary protein investigation (dipstick)**	11 14.9	28 28.9	37 72.5	65 85.5	31 93.9	15 93.9
**Uterine fundal height measurement (manual)**	37* 50.0	93 95.9	50 98.0	75 98.7	32 97.0	16 100.0
**Fetal heartbeat assessment**	1*1.4	59 60.8	48 94.1	74 97.4	32 97.0	16 100.0
**Fetal presentation assessment**	2* 2.7	15* 15.5	25* 49.0	68 89.5	32 97.0	14 87.5
**Prescription for folic acid supplements**	66 89.2	93* 95.9	1* 2	1* 1.3	0* 0.0	0* 0.0
**Prescription for folic acid and iron supplements**	11* 14.9	93 95.9	48 94.1	74 97.4	31 93.9	16 100.0
**Prescription for calcium supplements**	1* 1.4	36* 37.1	46 90.2	69 90.8	30 90.9	15 93.8

***N*** *= number of women attending a routine appointment within a gestational window*, ***n*** *= number of women who received a service during a gestational window*, ***CI*** *= confidence interval*,

* *= a service was provided out of a gestational window recommended by the MoHP*.

The delivery of antenatal services out of routine appointments is presented in Table 6. Maternal height measurements and the first human immunodeficiency virus (HIV) status examination were received by 99.1% of women with gestational ages ≥14 weeks. Over 80% of these women also had their blood and rhesus group determined and received all other first-time investigations. Second-time investigations were less frequent, with the second ultrasound examination being the most common (64.2%) and the second microscopic urine examination being the least (0%). A similar pattern was seen for anti-tetanus immunisations, as 80.6% of women received their first immunisation, whereas 38.2% received their second. One woman out of 121 (0.8%, 95% CI: 0.1%– 4.5%) received all services delivered out of routine appointments (as outlined in Table 6) exactly as per the MoHP protocol.

**Table 6:** Delivery of antenatal services out of routine appointments.

Service	Service received
	n % (95% CI)
**Women with gestational ages ≥14 weeks (completed window <14 weeks) (N = 112)**	
Maternal height measured	111 99.1 (95.1–99.8)
Blood group determined	97 86.6 (79.1–91.7)
Rhesus group determined	98 87.5 (80.1–92.4)
Pelvic exam	46 41.1 (32.4–50.3)
Cervical smear	24 21.4 (14.8–29.9)
First haemoglobin measurement	100 89.3 (82.2–93.8)
First microscopic urine examination	92 82.1 (74.0–88.1)
First serum glucose measurement	98 87.5 (80.1–92.4)
First HIV status test	111 99.1 (95.1–99.8)
First syphilis status test	107 95.5 (90.0–98.1)
First ultrasound examination	92 82.1 (74.0–88.1)
**Women with gestational ages ≥22 weeks (completed window 14–21 weeks) (N = 91)**	
Dental examination	33 36.3 (27.1–46.5)
First anti-tetanus immunisation (N = 62)*	50 80.6 (69.1–88.6)
**Women with gestational ages ≥25 weeks (completed window 22–24 weeks) (N = 81)**	
Second ultrasound examination	52 64.2 (53.5–73.8)
Second anti-tetanus immunisation (N = 55)*	21 38.2 (26.5–51.4)
**Women with gestational ages** ≥**33 weeks (completed window 25–32 weeks) (N = 41)**	
Second serum glucose measurement	14 34.1 (21.6–49.5)
**Women with gestational ages** ≥**37 weeks (completed window 33–36 weeks) (N = 16)**	
Second haemoglobin measurement	9 56.3 (33.2–76.9)
Second microscopic urine examination	0 0.0 (0.0–19.4)
Second HIV status test	6 37.5 (18.5–61.4)
Second syphilis status test	3 18.8 (6.6–43.0)

***CI*** *= confidence interval*,

* *= N for anti-tetanus immunisations vary as women with previous history of immunisations were not applicable for this service*.

Of all 121 participants for which hand-held antenatal cards were examined, none (0%, 95% CI: 0% - 3.1%) received all antenatal services delivered within and after routine appointments (the combination of all services outlined in Tables 5 and 6) exactly as per the MoHP protocol.

### Delivery of information as part of ANC

**Fig 2:**
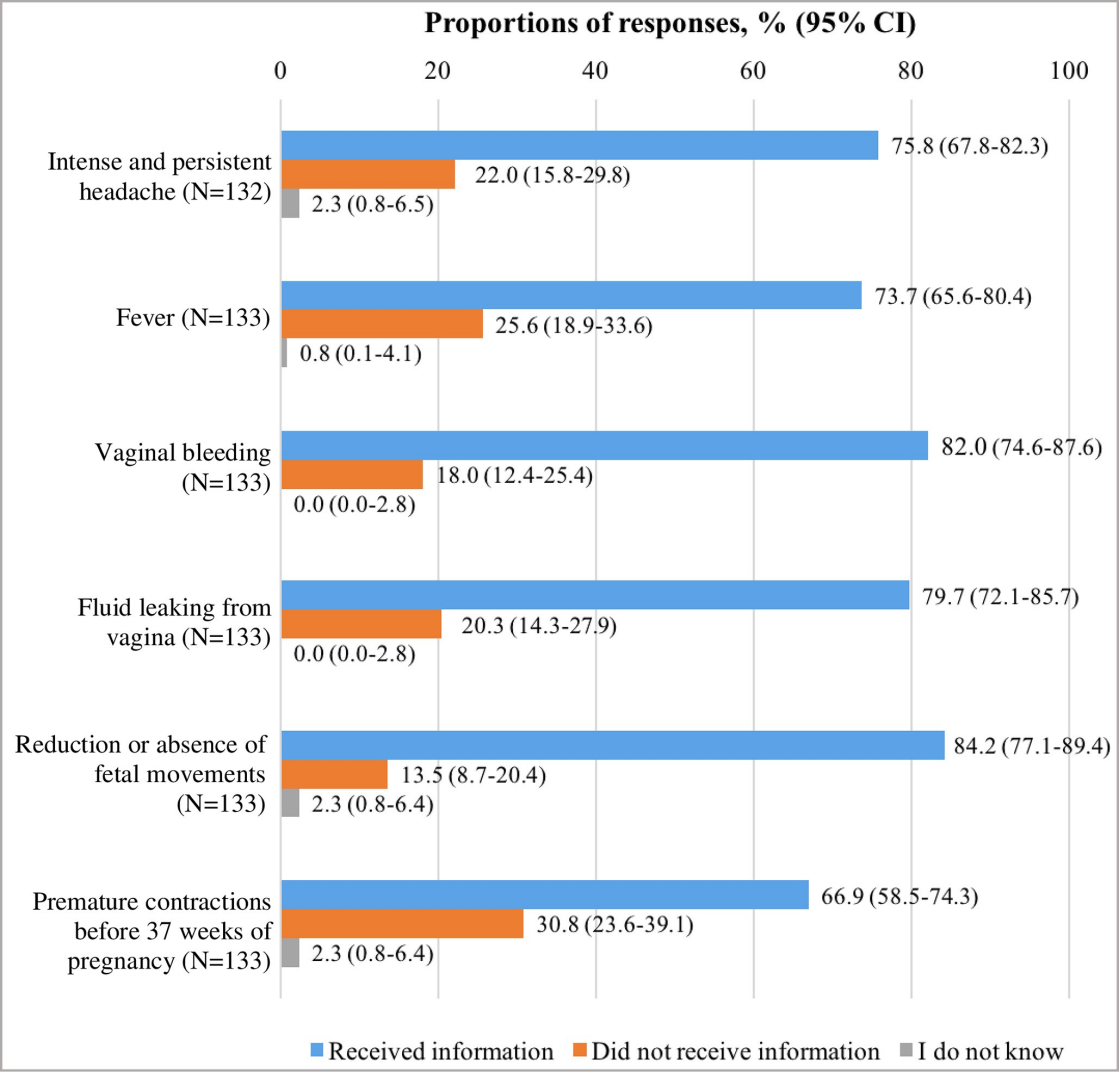
Reported delivery of information about identifying signs of complications of pregnancy. N = number of respondents for each question.

Fig 2 presents women’s reported delivery of information about identifying signs of complications of pregnancy. Approximately two-thirds or more of participants reported to have received information about identifying any of the six signs of complications. Women most commonly reported receiving information about identifying the reduction or absence of fetal movements (84.2%) and least commonly about identifying premature contractions (66.9%). Sixty-nine women (51.9%, 95% CI 43.4–60.7) reported to have received information about identifying all six signs of complications in pregnancy.

**Fig 3:**
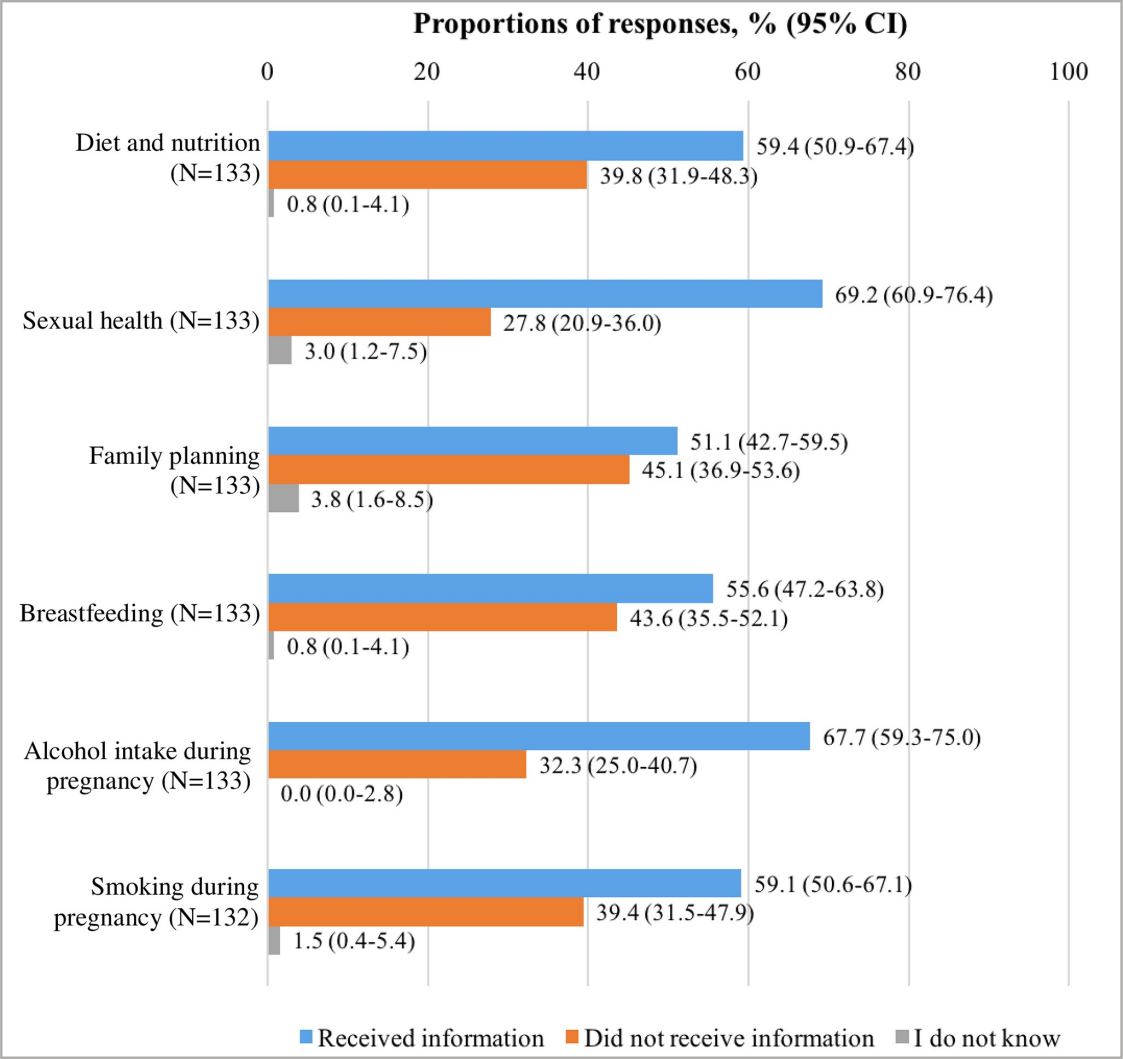
Reported delivery of information about health and lifestyle topics. N = number of respondents for each question.

Fig 3 presents the reported delivery of information about health and lifestyle topics. Over half of the questionnaire respondents reported to have received information about any of the six health and lifestyle topics investigated, with approximately two-thirds reporting that they received information about sexual health (69.2%) and alcohol intake during pregnancy (67.7%). Twenty-four women (18.0%, 95% CI 11.5–24.6) reported to have received information about all six health and lifestyle topics.

### Perceptions about ANC

Participants’ perceptions about their ANC are presented in Table 7. Most women found their hand-held antenatal cards useful (96.1%). Eighty-four (63.2%) respondents reported to have discussed their delivery plans with their antenatal provider(s) [S2 Table]. Out of these women, two-thirds (66.7%) were content with their involvement in the decisions made about their plans. Thirty-five (26.3%) reported that they received written pregnancy related health information and 95 (71.4%) reported that they did not [S3 Table]. Most women (97.1%) who reported to receive such information found it useful and most (88.4%) who reported that they did not receive such information would have liked to receive it.

**Table 7:** Women’s perceptions about their ANC.

Perception	n % (95 CI)
**Perception about hand-held antenatal card (N = 129)**	
I find my hand-held antenatal card useful	124 96.1 (91.2–98.3)
I do not find my hand-held antenatal card useful	1 0.8 (0.1–4.3)
I do not know	4 3.1 (1.2–7.7)
**Perception about the involvement in decisions made about delivery plans (N = 84)**^a^	
I am content with my involvement	56 66.7 (56.1–75.8)
I would like to be more involved	14 16.7 (10.2–26.1)
I would like to be less involved	0 0.0 (0.0–4.4)
I do not know	14 16.7 (10.2–26.1)
**Perception about written pregnancy related health information received (N = 35)**^b^	
I found this written information useful	34 97.1 (85.5–99.5)
I did not find this written information useful	0 0.0 (0.0–9.9)
I do not know	1 2.9 (0.5–14.5)
**Perception about receiving written pregnancy related health information (N = 95)**^c^	
I would like to receive such written information	84 88.4 (80.4–93.4)
I would not like to receive such written information	3 3.2 (1.1–8.9)
I do not know	8 8.4 (4.3–15.7)
**Perception about the duration of routine antenatal appointments (N = 133)**	
I am content with the duration of appointments	98 73.7 (65.6–80.4)
I would prefer longer appointments	10 7.5 (4.1–13.3)
I would prefer shorter appointments	18 13.5 (8.7–20.4)
I do not know	7 5.3 (2.6–10.5)
**Perception about the number of routine antenatal appointments received (N = 133)**	
I am content with the number of appointments received	104 78.2 (70.4–84.4)
I would prefer more appointments	16 12.0 (7.5–18.6)
I would prefer fewer appointments	4 3.0 (1.2–7.5)
I do not know	9 6.8 (3.6–12.4)
**Perception about the level of privacy experienced during routine appointments (N = 133)**	
I am content with the level of privacy I experienced	101 75.9 (68.0–82.0)
I would prefer more privacy	19 14.3 (9.3–21.2)
I would prefer less privacy	3 2.3 (0.8–6.4)
I do not know	10 7.5 (4.1–13.3)
**Overall satisfaction with ANC received (N = 133)**	
Very satisfied	28 21.1 (15.0–28.7)
Satisfied	90 67.7 (59.3–75.0)
Neutral	14 10.5 (6.4–16.9)
Unsatisfied	1 0.8 (0.1–4.1)
Very unsatisfied	0 0.0 (0.0–2.8)

***N*** *= number of respondents for each question*, ***CI*** *= confidence interval*,

^a^ *= out of questionnaire respondents who reported to have discussed their delivery plans with their antenatal provider(s)*,

^b^ *= out of questionnaire respondents who reported to have received such written information*,

^c^ *= out of questionnaire respondents who reported that they did not receive such written information*.

The median duration of routine appointments reported by 117 women (16 answered ‘I do not know’) was 20 minutes (Q1-Q3: 15–30). Three-quarters or more of women were content with the duration (73.7%) and number (78.2%) of routine appointments received, as well as the level of privacy experienced in routine appointments (75.9%). The majority of women were either satisfied (67.7%) or very satisfied (21.1%) with their ANC.

**Fig 4:**
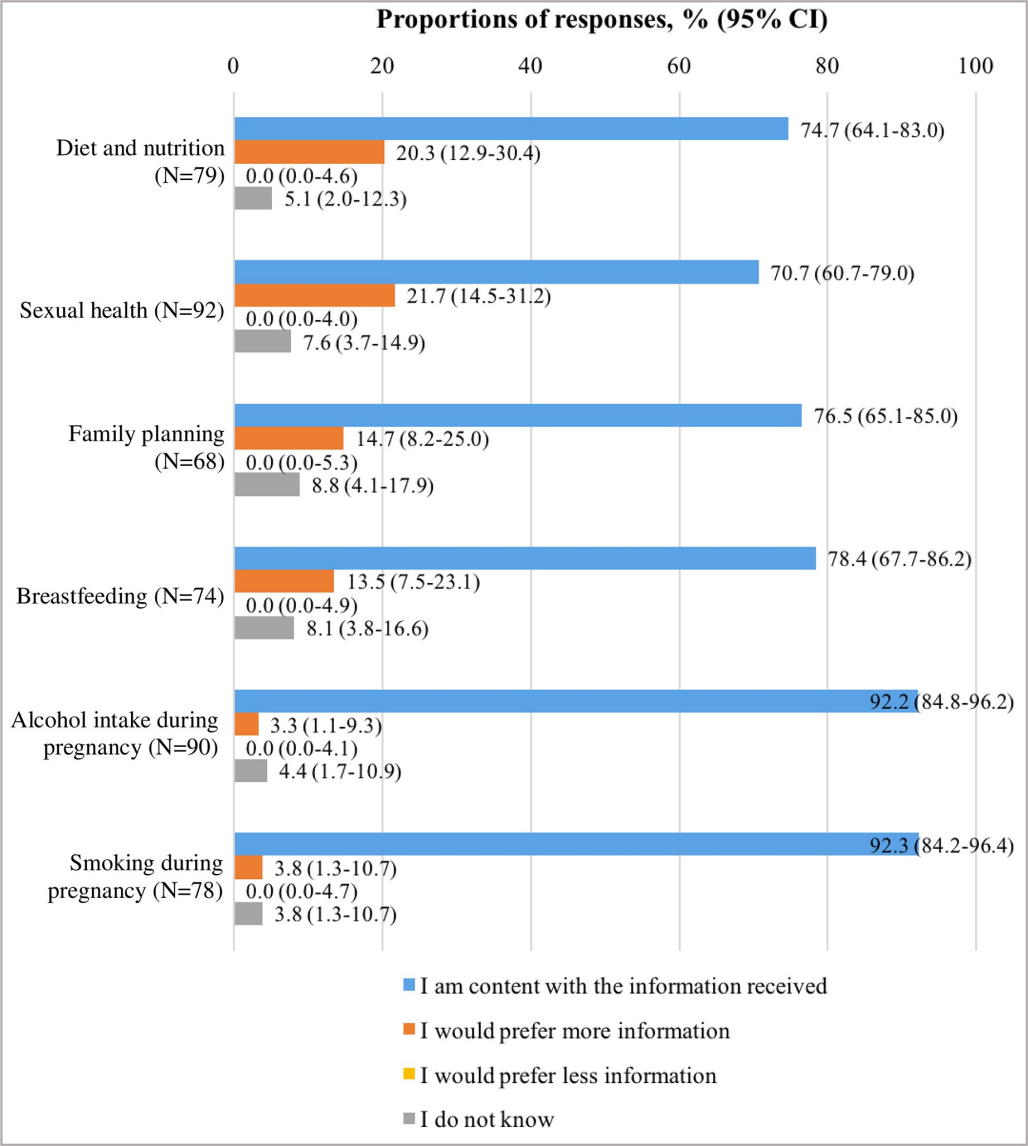
Women’s perceptions about information received about health and lifestyle topics. N = number of respondents for each question.

Women’s perceptions about health and lifestyle information received are presented in Fig 4. Over 70% of women who reported to have received information about any of the six health and lifestyle topics investigated were content with the information received, with over 90% being content with information received about alcohol intake (92.2%) and smoking during pregnancy (92.3%). None of the participants reported that they would prefer less information about any of the topics investigated.

## Discussion

### Key results

This is the first study to investigate the timing of antenatal appointments and the delivery of antenatal services at multiple gestational ages of pregnancy in the region of Loreto, Peru. No women received all of their antenatal services exactly as per the MoHP protocol. Services delivered out of routine appointments were received less frequently than those within routine appointments, particularly second-time services. Just over half of women initiated their ANC in their first trimester and fewer attended routine appointments according to the MoHP antenatal schedule. The majority of women received information about identifying signs of complications in pregnancy and health and lifestyle topics. Most women also had positive perceptions about their ANC, including high rates of satisfaction.

### Interpretation

The proportion of women initiating their ANC in their first trimester, although higher than previous reports from Iquitos (32.5%) and other developing countries, is lower than the national figure (79.8%) [6, 16, 32–38]. This could be due to the Amazonian culture in Iquitos, resulting in some women visiting practitioners of traditional Amazonian medicine before accessing ANC later in pregnancy [16, 39]. Women may also attend ANC late as a means of gaining a hand-held antenatal card before delivery, which allows access to free institutional deliveries in Iquitos [18, 40]. This has been observed in previous studies amongst women in Tanzania, South Africa, Malawi and Uganda [41–46]. Being unaware of pregnancy could also delay ANC, as previously demonstrated in South American women [39, 47, 48]. This was not explored as part of this study.

The high proportion of women attending their recommended number of appointments for their gestational age may be due to free access [2, 23, 28]. As well as women missing appointments, the lower proportion of women attending according to the MoHP schedule could be due to delayed initiation of ANC, resulting in an absence of appointments in earlier gestational windows. Community health promotion and education activities could be implemented within Iquitos to encourage more women to access ANC in their first trimester to meet WHO recommendations and encourage subsequent appointments to be attended according to the MoHP schedule [16, 40]. Trials of conditional cash transfer systems as an incentive for attending ANC have also been successful in improving rates of women attending antenatal appointments in Peru [49].

Encouraging women to attend according to the MoHP schedule may improve the frequency of women receiving all antenatal services as per the MoHP protocol, as the low frequency observed in this study was partly due to women missing appointments and having delayed initiation of ANC. Concerning the delivery of individual services, the re-usable nature of apparatus used to measure blood pressure and temperature in this low-resource setting may account for the high frequency of these services, in line with findings from other developing settings [2, 38]. The high frequency of most services received in routine appointments could also because the hand-held antenatal cards list the services to be provided, thus providing a checklist to be followed [36].

The low proportions of women having their urinary protein measured before 22 weeks reflects similar findings from health centres in Zambia [50]. This finding amongst women earlier in their pregnancy could be a means of saving resources of urine dipsticks, as one purpose of investigating urinary protein is to screen for pre-eclampsia, which classically presents after 20 weeks [1]. Urinary protein measurements are of particular importance in South America, where one-quarter of maternal deaths have been attributed to hypertensive disorders of pregnancy [51]. To meet WHO and MoHP, recommendations, urinary protein must be measured in every routine appointment as pre-eclampsia can rarely occur before 20 weeks and this investigation also assists in the early detection of pre-existing renal conditions [1, 52, 53].

In contrast, there was a high frequency of prescriptions for folic acid and iron supplements early in pregnancy. The timing of delivery of these services is important, as early consumption of folic acid supplements reduces the risk of neural tube defects in a fetus [54, 55]. Iron supplementation in early pregnancy also minimises the risk of pre-term delivery and the development of small for gestational age and low birth weight babies [56–58].

The timing of anti-tetanus immunisations is also important because women’s second anti-tetanus immunisations must be delivered at least two weeks before birth to ensure passive immunity for the prevention of neonatal tetanus [59, 60]. However, in this study, there was a low frequency of second anti-tetanus immunisations delivered to women who were not previously immunised. Furthermore, many women did not receive their second ultrasound examination by the end of their 24^th^ week of gestation. The MoHP recommends this scan between 22–24 weeks, as it is late enough in gestation to adequately visualise the development of fetal structures, but also early enough to identify and subsequently monitor and prepare for potential complications with the pregnancy and delivery, thus improving outcomes [61].

The lower frequency of second-time services received out of routine appointments could be due to women not being able return to healthcare centres to independently seek these services. This could be due to childcare, employment or inconvenient opening hours, as previously demonstrated in qualitative studies investigating barriers to accessing ANC amongst Peruvian women [23, 62]. Such services might be received more frequently if they were provided within routine appointments. This would eliminate the requirement for women to attend multiple times a week to complete their ANC, as observed in this study.

The proportions of women who received information about identifying the six complication signs investigated were higher than reports from other developing settings (Tanzania (3%) and the Gambia (20%)) [36, 63]. This could be because the signs are listed in the MoHP hand-held antenatal cards, which could allow for the delivery of information or reinforcement of information provided by clinicians [18]. However, higher proportions of women (>80%) reported to receive information about family planning, sexual health, breastfeeding and nutrition in Nigeria [32, 64]. Women in this study may have received information, but not perceived it to be delivered. As well as delivering information, clinicians must ensure that women comprehend and acknowledge any information received. As women in this study attended at least one routine appointment, this particularly applies to information about alcohol, smoking, sexual health and identifying complication signs, which should be discussed in the first appointment in the MoHP guidelines [18]. The WHO also recommends discussing smoking and alcohol at the first contact, regardless of the prevalence of smoking and alcohol drinking in the population area [1].

With respect to women’s perceptions, the findings in this population embody the WHO ‘positive pregnancy’ goal, women displayed a high rate of satisfaction (>80%) which reflects findings from both developed and developing countries [1, 32, 37, 64–66]. It is also promising that most women found their hand-held antenatal cards useful, unlike 93.3% of a sample of Nigerian women who preferred clinic-based notes, as the WHO promotes hand-held antenatal cards to encourage women to feel in control of their pregnancies [1, 64, 67]. However, as perceptions are influenced by social circles, culture and expectations, the high frequencies of satisfaction and contented women could be because delivered services met women’s personal requirements or expectations [31, 64].

Participants’ perceptions also highlighted areas for improvement. In-keeping with findings from qualitative research in Iquitos, antenatal care providers must ensure that all women feel involved in decision making about their birth [23]. Women in this study also expressed a desire for more written information to take home, reflecting the perceptions of other women from Iquitos [23]. More written pregnancy-related information could be added to women’s hand-held antenatal cards or provided in leaflets.

### Strengths and limitations

To our knowledge, this study is the first to investigate women’s perceptions about ANC and the delivery of antenatal services at multiple gestational ages in Peru. Medical records were examined to investigate the delivery of services, rather than questioning patients, thus eliminating recall bias for this data. However, the short data collection period accounted for a small sample size. The characteristics of non-responders were also not recorded, introducing the possibility of non-responder bias. Additionally, conducting interviews at the clinic at which ANC was received, in the presence of an interviewer, may have produced social-desirability bias resulting in the preponderance of women providing positive perceptions about their ANC, in addition to responding ‘I do not know’. The possibility of recall bias for self-reported information also exists. Although the study questionnaire was created with reference to previous questionnaires and WHO and MoHP antenatal guidelines, validating the study questionnaire was not within the scope of the study. This reduces the reliability of the study questionnaire. The use of structured questions also did not allow participants’ perceptions to be explored in-depth and women who had attended fewer antenatal contacts had less exposure to form their perceptions [32].

### Generalisability

As data were collected at one clinic in Iquitos, the findings of this study are not representative of the entire city. However, twelve other clinics in all districts of Iquitos were attended by some participants, extending the external validity of this study beyond that of the ANC received at the CSSJ. Additionally, the study findings lack external validity with respect to women under the age of 19 in Iquitos. This study also does not represent other cities and areas of Loreto, particularly rural parts of this Amazonian region.

## Conclusions

This study has expanded the limited existing research base concerning indicators of the quality of ANC received in a region with high MMR. The recent update in WHO antenatal guidelines also makes this research particularly relevant. To be more adherent to WHO and MoHP recommendations, community interventions could be implemented to encourage timely antenatal initiation and subsequent attendance. All recommended services could be offered within routine appointments to prevent women from returning independently to complete their ANC. Written pregnancy related health information could also be provided in women’s hand-held antenatal cards or leaflets.

## Supporting information

S1 Appendix Study questionnaire (English version). (DOCX)

S2 Appendix Study questionnaire (Peruvian Spanish version). (DOCX)

S1 Table Delivery of antenatal services during routine appointments. (DOC)

S2 Table Reported discussion about delivery plans with antenatal provider(s). (DOC)

S3 Table Reported delivery of written pregnancy related health information. (DOC)

## Abbreviations

ANC: Antenatal care
CI: Confidence interval
CSSJ: Centro de Salud, San Juan
HIV: Human immunodeficiency virus
IQR: Inter-quartile range
MMR: Maternal mortality ratio
MoHP: Ministry of Health, Peru
Q1-Q3: Quartile 1 –Quartile 3
WHO: World Health Organization
