# Supplementary material for: The timing and quality of antenatal care received by women attending a primary care centre in Iquitos, Peru: A facility exit survey
Source: PLoS One. 2020 Mar 5;15(3):e0229852. doi: 10.1371/journal.pone.0229852 (PMC7058332; doi:10.1371/journal.pone.0229852)
Supplement: S1 Appendix — (DOCX) [file pone.0229852.s001.docx]

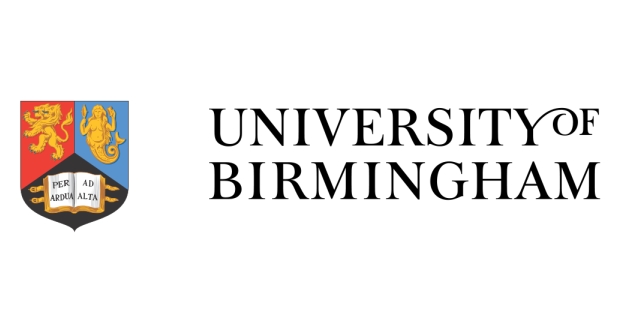


**Participant Questionnaire**

All of your responses will remain *strictly confidential*

The following questions are about ***ALL*** of the antenatal appointments you have attended in ***this pregnancy***.

**Questions about health information and advice in antenatal care**

1. During ***this pregnancy***, have you received any information about your diet and nutrition during pregnancy in your antenatal appointments?

No

I do not know

Yes

If you answered ***yes***, what is your opinion about the information

you received about diet and nutrition during pregnancy?

1. I would prefer less information
2. I am content with the information I received
3. I would prefer more information
4. I do not know
5. During ***this pregnancy***, have you received any information about your sexual health in your antenatal appointments (for example, about sexually transmitted infections)?

No

I do not know

Yes

If you answered ***yes***, what is your opinion about the information

you received about your sexual health?

1. I would prefer less information
2. I am content with the information I received
3. I would prefer more information
4. I do not know
5. During ***this pregnancy***, have you received any information about family planning/child spacing after your pregnancy in your antenatal appointments?

No

I do not know

Yes

If you answered ***yes***, what is your opinion about the information

you received about family planning/child spacing after your pregnancy?

1. I would prefer less information
2. I am content with the information I received
3. I would prefer more information
4. I do not know
5. During ***this pregnancy***, have you received any information about breastfeeding your new baby in your antenatal appointments?

No

I do not know

Yes

If you answered ***yes***, what is your opinion about the information

you received about breastfeeding your new baby?

1. I would prefer less information
2. I am content with the information I received
3. I would prefer more information
4. I do not know
5. During ***this pregnancy***, have you received any advice about drinking alcohol during pregnancy in your antenatal appointments?

I do not know

No

Yes

If you answered ***yes***, what is your opinion about

the advice you received about drinking alcohol

during pregnancy?

1. I would prefer less advice
2. I am content with the advice I received
3. I would prefer more advice
4. I do not know
5. During ***this pregnancy***, have you received any advice about smoking during pregnancy in your antenatal appointments?

No

I do not know

Yes

If you answered ***yes***, what is your opinion about

the advice you received about smoking during pregnancy?

1. I would prefer less advice
2. I am content with the advice I received
3. I would prefer more advice
4. I do not know
5. During ***this pregnancy***, did you receive any information about identifying any of the following ***potential*** complications of pregnancy?

I do not know

Yes

No

Severe or persistent headache

I do not know

Yes

Fever No

I do not know

Yes

No

Vaginal bleeding

I do not know

Yes

Fluid leaking (vaginal) No

I do not know

No

Yes

Decreased or absent

movements of your baby

Premature contractions (before

I do not know

Yes

37 weeks of pregnancy) No

20

**Questions about your opinions about your antenatal care**

1. What is your opinion about the number of antenatal appointments you have received during ***this pregnancy***?
2. I would prefer fewer appointments
3. I am happy with the number of appointments
4. I would prefer more appointments
5. I do not know
6. During ***this pregnancy***, how long do you usually spend with your antenatal clinician during your antenatal appointments (not including your waiting time)?

I do not know

Minutes

Hours

1. What is your opinion about the duration of the antenatal appointments that you have received for ***this pregnancy***?
2. I would prefer shorter appointments
3. I am content with the duration of my appointments
4. I would prefer longer appointments
5. I do not know

21

1. What is your opinion about the level of privacy you have experienced during your antenatal appointments for ***this pregnancy***?
   1. I would prefer more privacy during my antenatal appointments
   2. I am happy with the level of privacy that I have experienced
   3. I would prefer less privacy during my antenatal appointments
   4. I do not know
2. During ***this pregnancy***, did your antenatal clinician(s) discuss your delivery plans with you?

I do not know

No

Yes

If you answered ***yes*** to question 12, what is your opinion about your involvement in the decisions made about your delivery plans during ***this pregnancy***?

1. I would like to be more involved in the decisions made about my delivery plans
2. I would like to be less involved in the decisions made about my delivery plans
3. I am happy with my involvement in the decisions made about my delivery plans
4. I do not know
5. Do you have your own copy of your antenatal notes for ***this pregnancy***?

Yes, but I have lost them

Yes

No

If ***no***, would you like to have your own copy of your antenatal notes?

If you answered ***yes*** or ***yes, but I have lost them***,

do you/did you find having your

own notes helpful?

Yes No I do not know

Yes No I do not know

1. Did you receive any written information about looking after your health during pregnancy to take home with you after your antenatal appointments for ***this pregnancy***?

Yes No I do not know

If you answered ***yes*** to question 14, did you find it useful that you received written information about looking after your health during pregnancy to take home with you?

Yes No I do not know

If you answered ***no*** to question 14, would you have liked to receive written information about looking after your health during pregnancy to take home with you?

Yes No I do not know

1. Overall, how satisfied are you with the antenatal care you have received during ***this pregnancy***?
   1. Very satisfied
   2. Satisfied
   3. Neutral
   4. Unsatisfied
   5. Very unsatisfied

**Questions about you**

1. What is your age (in years)?
2. What is your marital status? Please circle one option only.

Single, never married

Married

Partnership

Separated

Widowed

Divorced

I would prefer not to say

1. What is your employment status?

Student

Unemployed

Employed

1. What is the highest level of education you have ***completed***? Please circle one option only.

University

Completed primary education

Other, please specify

Completed secondary education

*Tecnica* (between secondary and university)

1. How many times have you been pregnant ***before*** your current pregnancy (including miscarriages)?
2. How many children have you had ***before*** this pregnancy?
3. Is this health centre the only health centre you have attended for antenatal appointments during ***this pregnancy***?

Yes

No
