# Supplementary material for: The timing and quality of antenatal care received by women attending a primary care centre in Iquitos, Peru: A facility exit survey
Source: PLoS One. 2020 Mar 5;15(3):e0229852. doi: 10.1371/journal.pone.0229852 (PMC7058332; doi:10.1371/journal.pone.0229852)
Supplement: S2 Appendix — (DOCX) [file pone.0229852.s002.docx]

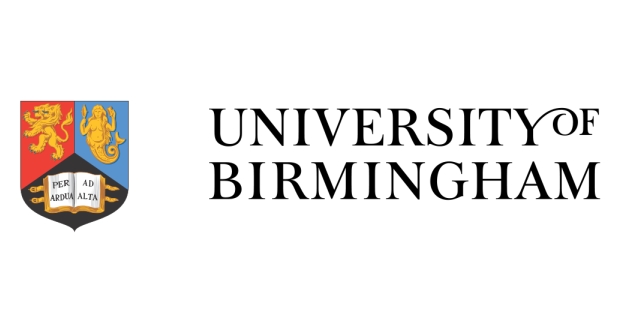


**Cuestionario para la Participante**

Todas sus respuestas permanencerán en *estricta confidencialidad*

Las suguentes preguntas son sobre ***TODAS*** las citas prenatales a las que usted ha asistido en ***este embarazo.***

**Preguntas acerca de la información y asesoramiento sobre salud en la atención prenatal**

1. Durante ***este embarazo***, ¿Ha recibido información acerca de su dieta y nutrición en sus citas prenatales?

No

No sé

Si

Si su respuesta es ***si***, ¿Cuál es su opinion sobre la información

recibida acerca de su deita y nutrición durante este embarazo?

1. Hubiera deseado menos información
2. Estoy contenta con la información que recibi
3. Hubiera desado mas información
4. No sé
5. Durante ***este embarazo***, ¿Ha recibido información acerca de su salud sexual en sus citas prenatales?

No

No sé

Si

Si su respuesta es ***si***, ¿Cuál es su opinion sobre la información

recibida acerca de su salud sexual durante este embarazo?

1. Hubiera deseado menos información
2. Estoy contenta con la información que recibi
3. Hubiera desado mas información
4. No sé
5. Durante ***este embarazo***, ¿Ha recibido información acerca de planificación familiar/espaciamiento entre el Nacimiento de los hijos después de su embaraxo en sus citas prenatales?

No

No sé

Si

Si su respuesta es ***si***, ¿Cuál es su opinion sobre la información

recibida acerca de planificación familiar/espaciamiento entre el

nacimiento de los hijos durante este embarazo?

1. Hubiera deseado menos información
2. Estoy contenta con la información que recibi
3. Hubiera desado mas información
4. No sé
5. Durante ***este embarazo***, ¿Ha recibido información acerca de amamantar a su neuvo bebé en sus citas prenatales?

No

No sé

Si

Si su respuesta es ***si***, ¿Cuál es su opinion sobre la información

recibida acerca de amamantar a su neuvo bebé durante

este embarazo?

1. Hubiera deseado menos información
2. Estoy contenta con la información que recibi
3. Hubiera desado mas información
4. No sé
5. Durante ***este embarazo***, ¿Ha recibido consejo acerca de tomar alcohol durante el embarazo en sus citas prenatales?

No

No sé

Si

Si su respuesta es ***si***, ¿Cuál es su opinion acerca del consejo

que recibió acerca de tomar alcohol durante el embarazo?

1. Hubiera deseado menos información
2. Estoy contenta con la información que recibi
3. Hubiera desado mas información
4. No sé
5. Durante ***este embarazo***, ¿Ha recibido consejo acerca de fumar durante el embarazo en sus citas prenatales?

No

No sé

Si

Si su respuesta es ***si***, ¿Cuál es su opinion acerca del consejo

que recibió acerca de fumar durante el embarazo?

1. Hubiera deseado menos información
2. Estoy contenta con la información que recibi
3. Hubiera desado mas información
4. No sé
5. Durante ***este embarazo***, ¿Recibió información para identificar cualquiera de las siguintes ***potenciales*** complicaciones del embarazo?

Si

No

No sé

Dolor de cabeza intenso o persistente

Si No sé

No

Fiebre

No sé

Si

No

Sangrado vaginal

No sé

Si

Salida de liquid por su vagina No

No sé

No

Si

Disminución o asusencia de los movimientos de su bebé

Contracciones prematuras (antes

No sé

Si

de las 37 semanas del embarazo) No

20

**Preguntas sobre su opinion de la atención prenatal**

1. ¿Cuál es su opinion sobre el número de citas prenatales que ha recibido durante ***este embarazo***?
2. Preferiría menos citas
3. Estoy contenta con el número de citas
4. Preferiría más citas
5. No sé
6. Durante ***este embarazo***, ¿Cuánto tiempo se demora normalmente con el medico que la atiende durante sus citas prenatales (sin incluir el tiempo de espera)?

No sé

Minutes

Horas

1. ¿Cuál es su opinion sobre la duración de las citas prenatales que ha recibido para ***este embarazo***?
2. Preferiría citas mas cortas
3. Estoy contenta con la duración de mis citas
4. Preferiría citas más largas
5. No sé
6. ¿Cuál es su opinion acerca del nivel de privacidad que ha experimentado durante sus citas prenatales durante ***este embarazo***?
7. Preferiría mas privacidad durante mis citas prenatales
8. Estoy contenta con el nivel de privacidad que he experimentado
9. Preferiría menos privacidad durante mis citas prenatales
10. No sé

21

1. Durante ***este embarazo***, ¿Conversó su medico prenatal con uested acerca de los planes para el parto?

Si

No sé

No

Si respondió ***si*** a la pregunta 12, ¿Cuál es su opinion acerca de su involuncramiento en las decisions tomadas acerca de sus planes de parto durante ***este embarazo***?

1. Me gustaría estar más involucrada en las decisions tomadas acerca de mis plammes de parto
2. Me gustaría estar menos involuncrada en las decisions tomadas acerca de mis planes de parto
3. Estoy contenta con mi participación en las decisions tomadas acerca de mis planes de parto
4. No sé
5. ¿Tiene su propria copia de sus registros prenatales para ***este embarazo***?

Si, pero los perdi

Si

No

Si es ***no***, ¿Le gustaría tener su propia copia de sus registros prenatales?

Si respondió si o si, pero los perdi, ¿Piensa que fue

útil tener esta copia de sus registros prenatales?

Si No No sé

Si No No sé

1. ¿Recibió alguna información escrita acerca del cuidado de su salud durante su embaraxo para llevar a su casa despuës de sus citas prenatales para ***este embarazo***?

Si No No sé

Si respondió ***si***  a la pregunta 14, ¿Piensa que es útil recibir información escrita acera del cuidado de sui salud durante su embarazo para llevar a su casa?

Si No No sé

Si respondió ***no***  a la pregunta 14, ¿Piensa que le hubiese gustado recibir información escrita acerca del guidado de su salud para llebar a su casa?

Si No No sé

1. De manera general, ¿Cuán satisfecha se siente con la atención prenatal que ha recibido durante ***este embarazo***?
   1. Muy satifecha
   2. Satisfecha
   3. Neutral
   4. Insatisfecha
   5. Muy insatisfecha

**Preguntas acerca de usted**

1. ¿Cual es su dad (en años?)
2. ¿Cuál es su estado marital? Por favour, coloque un circula solo en una opción?

Soltera, nunca me casé

Casada

Conviviente

Separada

Viuda

Divorciada

Preferiría no decirlo

1. ¿Cual es su situación laboral?

Estudiante

Desempleada

Empleada

1. ¿Cuál es el nivel de educación más alto que usted ha completado? Por favour, coloque un círcuolo solo en una opicón

Universidad

Primaria completa

Otros, por favour especificar

Secondaría completa

Tecnico superior

1. ¿Cuántas veces ha estado embarazada ***antes*** del actual embarazo (incluyendo pérdidas)?

1. ¿Cuántos hijos ha tenido ***antes*** de este embarazo?
2. ¿Es este centro de salud el único al que usted ha asistido para sus citas prenatales durante ***este embarazo***?

Si

No
