# Supplementary material for: The timing and quality of antenatal care received by women attending a primary care centre in Iquitos, Peru: A facility exit survey
Source: PLoS One. 2020 Mar 5;15(3):e0229852. doi: 10.1371/journal.pone.0229852 (PMC7058332; doi:10.1371/journal.pone.0229852)
Supplement: S1 Table — (DOC) [file pone.0229852.s003.doc]

**S3 Table: Delivery of antenatal services during routine appointments.**

| **Service** | **Gestational windows in which routine appointments were attended** | | | | | | | | | | | |
| --- | --- | --- | --- | --- | --- | --- | --- | --- | --- | --- | --- | --- |
|  | **<14 weeks (N=74)** | | **14-21 weeks (N=97)** | | **22-24 weeks (N=51)** | | **25-32 weeks (N=76)** | | **33-36 weeks (N=33)** | | **37-40 weeks (N=16)** | |
|  | **n** | **% (95% CI)** | **n** | **% (95% CI)** | **n** | **% (95% CI)** | **n** | **% (95% CI)** | **n** | **% (95% CI)** | **n** | **% (95% CI)** |
| **Maternal weight measurement** | | | | | | | | | | | | |
| ***Received*** | 74 | 100.0 (95.1-100.0) | 97 | 100.0 (96.2-100.0) | 51 | 100.0 (93.0-100.0) | 76 | 100.0 (95.2-100.0) | 33 | 100.0 (89.6-100.0) | 16 | 100.0 (80.6-100.0) |
| ***Not received*** | 0 | 0.0 (0.0-4.9) | 0 | 0.0 (0.0-3.8) | 0 | 0.0 (0.0-7.0) | 0 | 0.0 (0.0-4.8) | 0 | 0.0 (0.0-10.4) | 0 | 0.0 (0.0-19.4) |
| **Blood pressure measurement** | | | | | | | | | | | | |
| ***Received*** | 74 | 100.0 (95.1-100.0) | 97 | 100.0 (96.2-100.0) | 51 | 100.0 (93.0-100.0) | 76 | 100.0 (95.2-100.0) | 33 | 100.0 (89.6-100.0) | 16 | 100.0 (80.6-100.0) |
| ***Not received*** | 0 | 0.0 (0.0-4.9) | 0 | 0.0 (0.0-3.8) | 0 | 0.0 (0.0-7.0) | 0 | 0.0 (0.0-4.8) | 0 | 0.0 (0.0-10.4) | 0 | 0.0 (0.0-19.4) |
| **Pulse measurement** | | | | | | | | | | | | |
| ***Received*** | 74 | 100.0 (95.1-100.0) | 97 | 100.0 (96.2-100.0) | 51 | 100.0 (93.0-100.0) | 76 | 100.0 (95.2-100.0) | 33 | 100.0 (89.6-100.0) | 16 | 100.0 (80.6-100.0) |
| ***Not received*** | 0 | 0.0 (0.0-4.9) | 0 | 0.0 (0.0-3.8) | 0 | 0.0 (0.0-7.0) | 0 | 0.0 (0.0-4.8) | 0 | 0.0 (0.0-10.4) | 0 | 0.0 (0.0-19.4) |
| **Temperature measurement** | | | | | | | | | | | | |
| ***Received*** | 74 | 100.0 (95.1-100.0) | 97 | 100.0 (96.2-100.0) | 51 | 100.0 (93.0-100.0) | 76 | 100.0 (95.2-100.0) | 33 | 100.0 (89.6-100.0) | 16 | 100.0 (80.6-100.0) |
| ***Not received*** | 0 | 0.0 (0.0-4.9) | 0 | 0.0 (0.0-3.8) | 0 | 0.0 (0.0-7.0) | 0 | 0.0 (0.0-4.8) | 0 | 0.0 (0.0-10.4) | 0 | 0.0 (0.0-19.4) |
| **Urinary protein investigation (dipstick)** | | | | | | | | | | | | |
| ***Received*** | 11 | 14.9 (8.5-24.7) | 28 | 28.9 (20.8-38.6) | 37 | 72.5 (59.1-82.9) | 65 | 85.5 (75.9-91.7) | 31 | 93.9 (80.4-98.3) | 15 | 93.9 (71.7-98.9) |
| ***Not received*** | 63 | 85.1 (75.3-91.5) | 69 | 71.1 (61.4-79.2) | 14 | 27.5 (17.1-40.9) | 11 | 14.5 (8.3-24.1) | 2 | 6.1 (1.7-19.6) | 1 | 6.3 (1.1-28.3) |
| **Uterine fundal height measurement (manual)** | | | | | | | | | | | | |
| ***Received*** | 37***** | 50.0 (38.9-61.1) | 93 | 95.9 (89.9-98.4) | 50 | 98.0 (89.7-99.7) | 75 | 98.7 (92.9-99.8) | 32 | 97.0 (84.7-99.5) | 16 | 100.0 (80.6-100.0) |
| ***Not received*** | 37 | 50.0 (38.9-61.1) | 4 | 4.1 (1.6-10.1) | 1 | 2.0 (0.3-10.3) | 1 | 1.3 (0.2-7.1) | 1 | 3.0 (0.5-15.3) | 0 | 0.0 (0.0-19.4) |
| **Fetal heartbeat assessment** | | | | | | | | | | | | |
| ***Received*** | 1***** | 1.4 (0.2-7.3) | 59 | 60.8 (50.9-69.9) | 48 | 94.1 (84.1-98) | 74 | 97.4 (90.9-99.3) | 32 | 97.0 (84.7-99.5) | 16 | 100.0 (80.6-100.0) |
| ***Not received*** | 73 | 98.6 (92.7-99.8) | 38 | 39.2 (30.1-49.1) | 3 | 5.9 (2.0-15.9) | 2 | 2.6 (0.7-9.1) | 1 | 3.0 (0.5-15.3) | 0 | 0.0 (0.0-19.4) |
| **Fetal presentation assessment** | | | | | | | | | | | | |
| ***Received*** | 2***** | 2.7 (0.7-9.3) | 15***** | 15.5 (9.6-24.0) | 25***** | 49.0 (35.9-62.3) | 68 | 89.5 (80.6-94.6) | 32 | 97.0 (84.7-99.5) | 14 | 87.5 (64.0-96.5) |
| ***Not received*** | 72 | 97.3 (90.7-99.3) | 82 | 84.5 (76.0-90.4) | 26 | 51.0 (37.7-64.1) | 8 | 10.5 (5.4-19.4) | 1 | 3.0 (0.5-15.3) | 2 | 12.5 (3.5-36.0) |
| **Prescription for folic acid supplements** | | | | | | | | | | | | |
| ***Received*** | 66 | 89.2 (80.1-94.4) | 93***** | 95.9 (89.9-98.4) | 1***** | 2 (0.3-10.3) | 1***** | 1.3 (0.2-7.1) | 0***** | 0.0 (0.0-10.4) | 0***** | 0.0 (0.0-19.4) |
| ***Not received*** | 8 | 10.8 (5.6-19.9) | 4 | 4.1 (1.6-10.1) | 50 | 98 (89.7-99.7) | 75 | 98.7 (92.9-99.8) | 33 | 100.0 (89.6-100.0) | 16 | 100.0 (80.6-100.0) |
| **Prescription for folic acid and iron supplements** | | | | | | | | | | | | |
| ***Received*** | 11***** | 14.9 (8.5-24.7) | 93 | 95.9 (89.9-98.4) | 48 | 94.1 (84.1-98.0) | 74 | 97.4 (90.9-99.3) | 31 | 93.9 (80.4-98.3) | 16 | 100.0 (80.6-100.0) |
| ***Not received*** | 63 | 85.1 (75.3-91.5) | 4 | 4.1 (1.6-10.1) | 3 | 5.9 (2.0-15.9) | 2 | 2.6 (0.7-9.1) | 2 | 6.1 (1.7-19.6) | 0 | 0.0 (0.0-19.4) |
| **Prescription for calcium supplements** | | | | | | | | | | | | |
| ***Received*** | 1***** | 1.4 (0.2-7.3) | 36***** | 37.1 (28.2-47.0) | 46 | 90.2 (79.0-95.7) | 69 | 90.8 (82.2-95.5) | 30 | 90.9 (76.4-96.9) | 15 | 93.8 (71.7-98.9) |
| ***Not received*** | 73 | 98.6 (92.7-99.8) | 61 | 62.9 (53.0-71.8) | 5 | 9.8 (4.3-21.0) | 7 | 9.2 (4.5-17.8) | 3 | 9.1 (3.1-23.6) | 1 | 6.3 (1.1-28.3) |

**N** = number of women attending a routine appointment within a gestational window, **CI** = confidence interval, ***** = a service was provided out of a gestational window recommended by the MoHP.
