# Supplementary material for: The timing and quality of antenatal care received by women attending a primary care centre in Iquitos, Peru: A facility exit survey
Source: PLoS One. 2020 Mar 5;15(3):e0229852. doi: 10.1371/journal.pone.0229852 (PMC7058332; doi:10.1371/journal.pone.0229852)
Supplement: S2 Table — (DOC) [file pone.0229852.s004.doc]

**S4 Table:** Reported discussion about delivery plans with antenatal provider(s) (N=133).

| **Reported discussion** | **n** | **% (95% CI)** |
| --- | --- | --- |
| Discussed delivery plans | 84 | 63.2 (54.7-70.9) |
| Did not discuss delivery plans | 44 | 33.1 (25.7-41.5) |
| I do not know | 5 | 3.8 (1.6-8.5) |

***N*** *= number of respondents,* ***CI*** *= confidence interval.*
