# Supplementary material for: The timing and quality of antenatal care received by women attending a primary care centre in Iquitos, Peru: A facility exit survey
Source: PLoS One. 2020 Mar 5;15(3):e0229852. doi: 10.1371/journal.pone.0229852 (PMC7058332; doi:10.1371/journal.pone.0229852)
Supplement: S3 Table — (DOC) [file pone.0229852.s005.doc]

**S5 Table:** Reported delivery of written pregnancy related health information (N=133).

| **Reported delivery** | **n** | **% (95% CI)** |
| --- | --- | --- |
| Received information | 35 | 26.3 (19.6-34.4) |
| Did not receive information | 95 | 71.4 (63.2-78.4) |
| I do not know | 3 | 2.3 (0.8-6.4) |

***N*** *= number of respondents,* ***CI*** *= confidence interval.*
